# Central Adiposity and Anthropometric Indices as Gender-Specific Predictors of Systolic Blood Pressure in Young Adults

**DOI:** 10.7759/cureus.88961

**Published:** 2025-07-29

**Authors:** Narendra Prasad Tiwari, Rekha Jiwane, Sonal Gupta, Gourav S Raghuwanshi

**Affiliations:** 1 Department of Physiology, People's College of Medical Sciences and Research Centre, Bhopal, IND; 2 Department of Physiology, All India Institute of Medical Sciences, Bhopal, Bhopal, IND; 3 School of Electrical and Electronics Engineering, VIT Bhopal University, Bhopal, IND

**Keywords:** body mass index, central adiposity, gender differences, hypertension, medical students, systolic blood pressure, waist circumference, waist-to-hip ratio, young adults

## Abstract

Background

Hypertension is a rising concern among young adults, driven by sedentary lifestyles, academic stress, and poor dietary habits. Medical students, despite being a theoretically low-risk group, exhibit a notable prevalence of elevated blood pressure. Traditional measures such as body mass index (BMI) fail to capture fat distribution, a critical determinant of cardiovascular risk. This study investigates the association of anthropometric indices - BMI, waist circumference (WC), and waist-to-hip ratio (WHR) - with systolic blood pressure (SBP), emphasizing gender-specific patterns in a population of medical students.

Methods

A cross-sectional study was conducted among 250 medical students (175 males, 75 females) aged 17-25 years. Anthropometric measurements, including BMI, WC, and WHR, were recorded following standardized protocols. Blood pressure was measured using the auscultatory method, and elevated SBP (>120 mmHg) was defined per the 2017 American College of Cardiology (ACC) and American Heart Association (ACC/AHA) guidelines. Chi-square (χ²) tests were used to assess associations between categorized anthropometric indices and SBP, with p < 0.05 considered significant.

Results

Elevated SBP was observed in 24.0% of males (42 out of 175) and 25.3% of females (19 out of 75). Among males, significant associations were identified between elevated SBP and BMI (χ² = 13.097, p = 0.0003), WC (χ² = 10.075, p = 0.0015), and WHR (χ² = 8.701, p = 0.0032). In females, WC alone demonstrated a significant association with SBP (χ² = 6.329, p = 0.0119), while BMI (p = 0.1643) and WHR (p = 0.7844) did not. Comparative analysis highlighted BMI and WHR as stronger predictors of elevated SBP in males, whereas WC consistently emerged as the most significant predictor across both genders.

Conclusion

Central adiposity, as indicated by WC, is a pivotal predictor of SBP in young adults, reinforcing its clinical utility over BMI and WHR. Gender-specific trends highlight the pronounced role of BMI and WHR in males, while WC remains the sole significant predictor in females. These findings underscore the importance of incorporating WC measurements into routine screening protocols for early identification of at-risk individuals. Early, targeted interventions addressing central adiposity may play a crucial role in preventing the progression of hypertension and reducing cardiovascular morbidity in young adults.

## Introduction

Hypertension is a significant global health concern, affecting approximately 1.28 billion adults worldwide and serving as a leading risk factor for cardiovascular diseases, renal failure, and stroke [1]. The increasing prevalence of hypertension among young adults, particularly those aged 18-39, is alarming, with recent studies indicating a prevalence rate between 22.4% and 31.2% in this age group [2,3]. Early-onset hypertension is associated with a higher lifetime risk of cardiovascular events, underscoring the importance of early identification and management of modifiable risk factors [4].

Obesity is a well-established modifiable risk factor for hypertension. Traditional measures such as body mass index (BMI) are commonly used to assess obesity; however, BMI does not account for fat distribution, which is crucial in determining cardiovascular risk. Central adiposity, measured through indices such as waist circumference (WC) and waist-to-hip ratio (WHR), has been shown to be more strongly associated with hypertension and cardiovascular diseases [5]. A recent study found that central obesity is associated with an increased risk of hypertension, even among individuals with a normal BMI [6].

Gender differences play a significant role in the relationship between adiposity and hypertension. Men are more likely to accumulate visceral fat, which is metabolically active and contributes to higher blood pressure levels [7]. In contrast, women tend to accumulate subcutaneous fat, which has different metabolic implications. However, increased central adiposity in women is also associated with a higher risk of developing hypertension [8].

Young adults, including medical students, are increasingly susceptible to hypertension due to factors such as academic stress, sedentary lifestyles, and poor dietary habits [9]. Despite being in a theoretically low-risk group, studies have reported a notable prevalence of elevated blood pressure among medical students, with significant associations to stress, anxiety, and lifestyle factors [10,11].

Given the limitations of traditional anthropometric measures and the growing burden of hypertension in young adults, this study aims to evaluate the associations between BMI, WC, and WHR with systolic blood pressure (SBP) among medical students, with a focus on gender-specific patterns. Our previous study from the same cohort demonstrated gender-specific associations between body composition indices and SBP: fat mass index (FMI) was significantly associated with SBP in males, while fat-free mass index (FFMI) showed a stronger correlation in females [12]. This analysis extends the investigation to traditional anthropometric measures - BMI, WC, and WHR - to assess their predictive utility for SBP within the same population.

## Materials and methods

Study design and participants

This cross-sectional study was conducted on 250 medical students (175 males and 75 females) aged 17-25 years from MGM Medical College, Indore, India. Participants were selected through convenience sampling, and informed consent was obtained prior to data collection. The study was conducted over a period from May 2015 to August 2016. Ethical clearance was granted by the institutional ethics committee.

Anthropometric measurements

All measurements were conducted using calibrated instruments, following standardized protocols to ensure consistency and reliability.

Height Measurement

Height was measured to the nearest 0.1 cm using a wall-mounted stadiometer. During the measurement, participants stood barefoot and maintained contact with the stadiometer at their heels, buttocks, shoulders, and head.

Weight Measurement

Weight was recorded using an electronic weighing scale (Hanson Model H89DK; reliability coefficient r = 0.80), accurate to the nearest 0.5 kg. During the measurement, participants were barefoot and dressed in light clothing.

Body Mass Index (BMI)

BMI was calculated as follows: BMI (kg/m²) = Weight (kg) / (Height (m))². Participants were categorized into different groups based on BMI thresholds according to the World Health Organization (WHO) classification. The thresholds used were: a BMI of ≤24.99 kg/m² for normal weight and a BMI of ≥25.00 kg/m² for overweight. A BMI greater than 25 was selected to reflect increased metabolic and cardiovascular risk, aligning with WHO guidelines that define overweight as a BMI of ≥25 [13].

Waist and Hip Circumferences

WC was measured midway between the lowest rib and the iliac crest using a non-stretchable measuring tape, while hip circumference was measured at the widest part of the buttocks, with both measurements recorded to the nearest 0.1 cm.

In this study, WC thresholds of >90 cm for males and >80 cm for females were used as markers of abdominal obesity. These cut-off values align with the guidelines recommended by the International Diabetes Federation (IDF) for South Asian, Chinese, and Japanese populations, as detailed in the WHO report on WC and WHR [13].

Waist-to-Hip Ratio (WHR)

WHR was calculated as follows: WHR = Waist Circumference (cm) / Hip Circumference (cm). In this study, the thresholds for considering WHR as abnormal were set at >1.0 for males and >0.9 for females. These values are based on the WHO recommendations of >0.90 for males and >0.85 for females [13], while the National Institute of Diabetes and Digestive and Kidney Diseases (NIDDK) uses stricter cut-offs of ≥1.0 for males and ≥0.8 for females [14].

The chosen thresholds reflect the higher visceral adiposity and increased metabolic risks observed in South Asian populations [15], ensuring more effective identification of individuals at increased cardiovascular and metabolic risk.

Blood pressure measurement

Systolic blood pressure (SBP) was measured using a standard mercurial sphygmomanometer by the auscultatory method. To minimize circadian variations, measurements were taken at 12:30 PM following a 10-minute rest period. Three consecutive readings were recorded at one-minute intervals, and the average value was used for analysis. SBP was categorized as normal if ≤120 mmHg and elevated if >120 mmHg.

In this study, high SBP was defined as ≥120 mmHg, adhering to the 2017 American College of Cardiology (ACC) and American Heart Association (AHA) guidelines, which classify an SBP of 120-129 mmHg as elevated and indicative of increased cardiovascular risk [16].

Statistical analysis

Data were analyzed using Statistical Product and Service Solutions (SPSS, version 26; IBM SPSS Statistics for Windows, Armonk, NY). Descriptive statistics (mean ± standard deviation) were calculated for all variables. Anthropometric measures (BMI, WC, and WHR) and SBP were dichotomized into normal and elevated categories based on study-specific thresholds. The chi-square (χ²) test was used to evaluate associations between SBP and these categorized anthropometric measures, as it is appropriate for assessing relationships between categorical variables. Statistical significance was assessed using a two-tailed approach, with the threshold for significance set at p < 0.05. Associations with p < 0.05 were considered significant, while p < 0.001 were categorized as highly significant. All analyses were performed following standard statistical practices, and the results were interpreted accordingly.

## Results

Descriptive statistics

**Figure 1:**
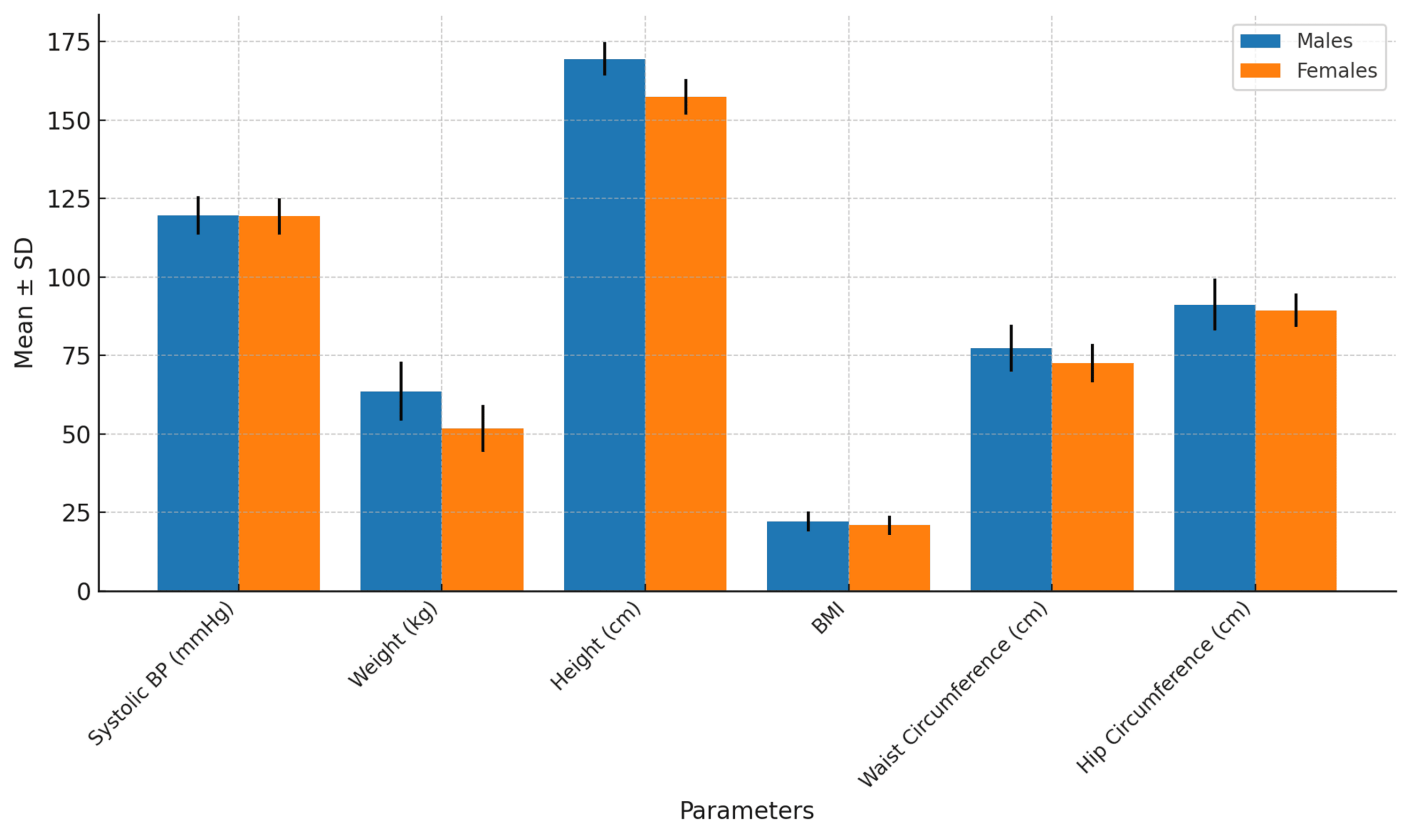
Descriptive characteristics of the study population Visual representation of the distribution of systolic blood pressure (SBP), weight, height, body mass index (BMI), waist circumference (WC), and hip circumference (HC) across genders (mean ± SD).

**Figure 2:**
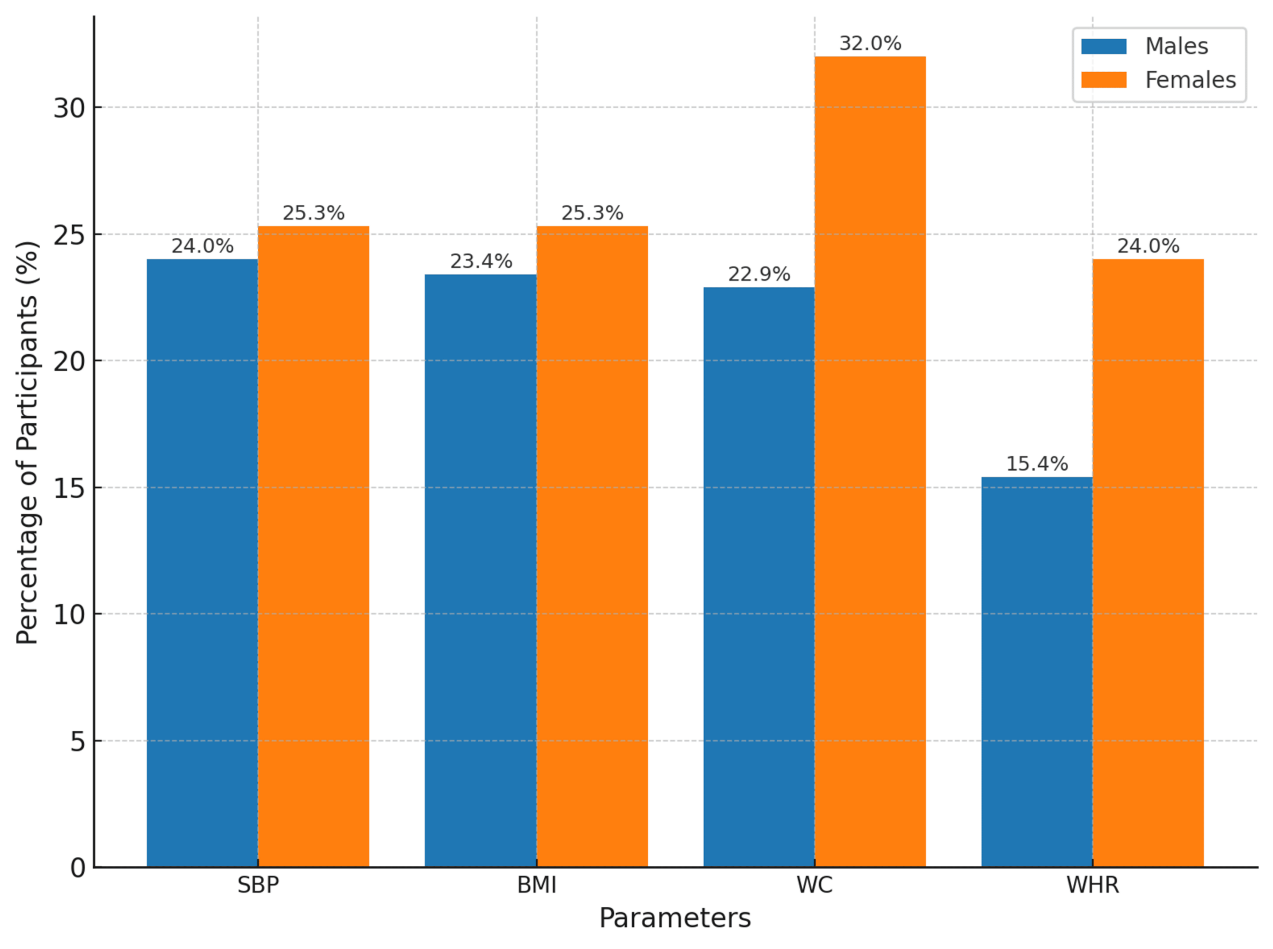
Proportion of participants exceeding study-specific thresholds for systolic blood pressure and anthropometric parameters Percentage of participants exceeding study-specific thresholds for systolic blood pressure (SBP), body mass index (BMI), waist circumference (WC), and waist-to-hip ratio (WHR), stratified by gender.

The descriptive characteristics of the study population (N = 250) are detailed in Table 1, which summarizes the mean and standard deviation values for SBP, weight, height, BMI, WC, and HC, stratified by gender. The graphical representation in Figure 1 provides a visual comparison of these parameters across genders, highlighting differences in anthropometric measures, such as weight, height, and WC. Furthermore, Figure 2 illustrates the proportion of participants exceeding study-specific thresholds for BMI, WC, and WHR, emphasizing notable gender-based disparities, such as the higher prevalence of elevated WC and WHR among females compared to males.

**Table 1:** Mean and standard deviation of measured parameters Data are presented as mean ± standard deviation (SD) for systolic blood pressure (SBP) and anthropometric parameters, stratified by gender.

Parameter	Total (N=250)	Males (N=175)	Females (N=75)
Systolic Blood Pressure (SBP, mmHg)	119.7 ± 5.9	119.7 ± 6.1	119.3 ± 5.7
Weight (kg)	60.1 ± 10.4	63.6 ± 9.4	51.8 ± 7.4
Height (cm)	165.8 ± 7.8	169.5 ± 5.4	157.4 ± 5.6
Body Mass Index (BMI)	21.7 ± 3.1	22.1 ± 3.1	20.9 ± 3.03
Waist Circumference (WC, cm)	75.9 ± 7.4	77.3 ± 7.5	72.6 ± 6.1
Hip Circumference (HC, cm)	90.6 ± 7.4	91.2 ± 8.3	89.4 ± 5.4

Summary

SBP, BMI, WC, and WHR exhibited variability across genders. Elevated SBP (>120 mmHg) was observed in 24.0% of males and 25.3% of females. Elevated BMI (≥25) was noted in 23.4% of males and 25.3% of females. However, elevated WC was more common in females (32.0%) compared to males (22.9%), as was elevated WHR (females: 24.0%, males: 15.4%).

Association between anthropometric measures and SBP

The chi-square (χ²) test was used to evaluate the association between SBP and categorized anthropometric measures (BMI, WC, and WHR). Anthropometric measures and SBP were dichotomized into normal and elevated categories based on study-specific thresholds. The findings are presented below.

Association Between BMI and SBP

The association between BMI and SBP is shown in Table 2.

**Table 2:** Association between BMI and systolic blood pressure (SBP) in males and females Values are represented as absolute numbers (N) of participants within each BMI and SBP category. Associations were assessed using the chi-square (χ²) test. A p-value < 0.05 was considered statistically significant; p < 0.001 was considered highly significant.

Group	SBP ≤ 120 mmHg	SBP > 120 mmHg	Chi-square (χ²)	p-value	Significance
Males (N=175)			13.097	0.0003	Highly Significant
Normal BMI (≤24.99)	111	23			
High BMI (>25)	22	19			
Females (N=75)			1.935	0.1643	Not Significant
Normal BMI (≤24.99)	46	10			
High BMI (>25)	12	7			

Key Findings

The association between BMI and SBP demonstrated notable gender-specific trends. Among males, 46.3% (19 out of 41) of participants with elevated BMI (>25) exhibited elevated SBP (>120 mmHg), compared to 17.2% (23 out of 134) of those with normal BMI (≤24.99). This association was highly significant, as indicated by the chi-square test (χ² = 13.097, p = 0.0003). In contrast, 36.8% (seven out of 19) of females with elevated BMI had elevated SBP, compared to 17.9% (10 out of 56) of those with normal BMI; however, this relationship was not statistically significant (χ² = 1.935, p = 0.1643).

Association Between WC and SBP

The relationship between WC and SBP is detailed in Table 3.

**Table 3:** Association between WC and SBP in males and females Values are represented as absolute numbers (N) of participants within each waist circumference (WC) and SBP category. The chi-square (χ²) test was used for analysis. A p-value < 0.05 was considered statistically significant; p < 0.001 was considered highly significant.

Group	SBP ≤ 120 mmHg	SBP > 120 mmHg	Chi-square (χ²)	p-value	Significance
Males (N=175)			10.075	0.0015	Significant
WC ≤ 90 cm	114	21			
WC > 90 cm	25	15			
Females (N=75)			6.329	0.0119	Significant
WC ≤ 80 cm	43	8			
WC > 80 cm	13	11			

Key Findings

The association between WC and SBP was found to be significant for both males and females. Among males, 37.5% (15 out of 40) of participants with elevated WC (>90 cm) exhibited elevated SBP (>120 mmHg), compared to 15.5% (21 out of 135) of those with normal WC (≤90 cm). This relationship was statistically significant (χ² = 10.075, p = 0.0015). Similarly, in females, 45.8% (11 out of 24) of participants with elevated WC (>80 cm) had elevated SBP, compared to 15.7% (8 out of 51) of those with normal WC (≤80 cm), with the chi-square test confirming statistical significance (χ² = 6.329, p = 0.0119).

Association Between WHR and SBP

The results for WHR and SBP are presented in Table 4.

**Table 4:** Association between WHR and SBP in males and females Values are represented as absolute numbers (N) categorized by waist-to-hip ratio (WHR) and SBP levels. The chi-square (χ²) test was used to assess associations. A p-value < 0.05 was considered statistically significant; p < 0.001 was considered highly significant.

Group	SBP ≤ 120 mmHg	SBP > 120 mmHg	Chi-square (χ²)	p-value	Significance
Males (N=175)			8.701	0.0032	Significant
WHR ≤ 1	119	29			
WHR > 1	14	13			
Females (N=75)			0.075	0.7844	Not Significant
WHR ≤ 0.9	43	14			
WHR > 0.9	13	5			

Key Findings

The WHR demonstrated a significant association with SBP in males but not in females. Among males, 48.1% (13 out of 27) of participants with elevated WHR (>1) exhibited elevated SBP (>120 mmHg), compared to 19.6% (29 out of 148) of those with normal WHR (≤1). This relationship was statistically significant (χ² = 8.701, p = 0.0032). Conversely, no significant association was observed between WHR and SBP in females (χ² = 0.075, p = 0.7844). Among females with elevated WHR (>0.9), 27.8% (five out of 18) exhibited elevated SBP, compared to 24.6% (14 out of 57) of those with normal WHR (≤0.9).

Comparison of Predictors

The associations between BMI, WC, and WHR with elevated SBP revealed distinct gender-specific patterns. Among males, BMI demonstrated the strongest association with elevated SBP (χ² = 13.097, p = 0.0003), followed by WC (χ² = 10.075, p = 0.0015) and WHR (χ² = 8.701, p = 0.0032). In contrast, among females, WC emerged as the only significant predictor of elevated SBP (χ² = 6.329, p = 0.0119), while BMI (p = 0.1643) and WHR (p = 0.7844) did not show statistically significant associations. These results underscore the differing predictive utility of these anthropometric measures across genders.

Key Findings

The results reveal gender-specific patterns in the association between anthropometric measures and elevated SBP. In males, BMI has the most prominent association with elevated SBP, followed closely by WC and WHR. For females, WC alone demonstrates a significant association with elevated SBP.

## Discussion

This study highlights the significant associations between SBP and anthropometric indices, such as BMI, WC, and WHR, among medical students aged 17-25 years, with distinct gender-specific trends observed. Notably, WC emerged as a significant predictor of elevated SBP in both males and females, while BMI and WHR showed strong associations with SBP predominantly in males. These findings emphasize the critical role of central adiposity in hypertension risk among young adults, providing important insights into the physiological and gender-specific determinants of blood pressure regulation.

Our earlier study specifically examined the associations of body composition indices, FMI, and FFMI with SBP in the same cohort and identified significant gender-specific trends [12]. The FMI had a stronger association with SBP in males, while the FFMI correlated more significantly in females. In contrast, the current study highlights the predictive value of traditional anthropometric indices, particularly WC, which emerged as a consistent predictor across both genders. The differences observed reflect the complementary but distinct physiological roles of overall body composition versus central adiposity in blood pressure regulation.

Relevance and implications of findings

The significant association between elevated BMI and SBP in males, as indicated by the chi-square (χ²) test, underscores the role of overall adiposity in driving hypertension in this group [17]. Elevated BMI reflects an increased total fat mass, which has been associated with heightened sympathetic nervous system (SNS) activity, systemic inflammation, and insulin resistance, all contributing to elevated vascular tone and blood pressure [18,19]. These mechanisms align with findings from earlier studies, which identified BMI as a strong determinant of hypertension in young males [20].

Conversely, BMI did not significantly correlate with SBP in females, reflecting the nuanced role of body composition in females [17]. Subcutaneous fat, the predominant fat type in women, has less metabolic activity and thus may exert a reduced impact on vascular resistance and SNS activation compared to visceral fat, which is more prevalent in males [8].

The significant association of WC with elevated SBP in both males and females highlights the role of central adiposity in blood pressure regulation [17]. WC, a marker of visceral fat, is a better predictor of hypertension than BMI due to its closer association with insulin resistance, renin-angiotensin-aldosterone system (RAAS) activation, and pro-inflammatory cytokine release [6]. These physiological pathways increase vascular resistance and sodium retention, driving blood pressure elevation [19]. WC alone has been shown to correlate more strongly with the amount of visceral fat compared to the waist-to-hip ratio, making it a more robust indicator of central adiposity and associated health risks [7]. Similar results have been reported in young adults, where WC was a stronger correlate of SBP than BMI, particularly in populations with lower overall BMI but higher abdominal adiposity [6].

Interestingly, the WHR significantly predicted SBP in males but not in females. This aligns with the understanding that visceral fat deposition, reflected by the WHR, is more pronounced and metabolically active in males. The lack of association in females may be due to their relatively higher proportion of subcutaneous fat, which is less closely linked to vascular dysfunction [8]. Additionally, the protective effects of estrogen, which enhance endothelial function and modulate inflammation, may partially offset the impact of central adiposity in premenopausal females [8,21].

Comparative insights

While this study corroborates earlier findings on the role of central adiposity in hypertension, certain differences warrant attention. For instance, the stronger association of WHR with SBP in males in our study contrasts with findings from some studies conducted in older cohorts, where WC was the dominant predictor across genders [6]. This variation may reflect age-related changes in fat distribution, with WHR becoming less indicative of visceral adiposity in older individuals due to increased subcutaneous fat deposition.

The absence of significant associations between WHR and SBP in females is consistent with previous research suggesting a threshold effect, wherein central adiposity must exceed certain levels to exert a measurable impact on blood pressure [22,23]. Our findings extend this understanding by demonstrating that WC, rather than WHR, is a more sensitive marker of elevated SBP in young females, particularly in populations with lower baseline visceral fat.

Physiological mechanisms

Central adiposity exacerbates hypertension through multiple pathways, including increased SNS activity, RAAS activation, and impaired nitric oxide-mediated vasodilation [6,19]. The strong associations observed in this study reflect these mechanisms, particularly the pro-inflammatory effects of visceral fat, which elevate vascular resistance and promote endothelial dysfunction. Insulin resistance, often linked to abdominal obesity, further compounds these effects by increasing sodium retention and reducing renal filtration rates [6].

Limitations and future directions

This study's cross-sectional design limits causal inferences between adiposity and blood pressure. Additionally, the homogeneous sample of medical students restricts the generalizability of findings to broader populations. Future research should employ longitudinal designs to explore temporal changes in adiposity and SBP, including the impact of lifestyle interventions. Expanding the cohort to include diverse ethnic, socioeconomic, and age groups would enhance the applicability of results. Incorporating advanced imaging techniques to assess visceral and subcutaneous fat distribution could also provide deeper insights into the mechanisms linking adiposity and hypertension.

## Conclusions

This study highlights the pivotal role of central adiposity, particularly WC, as a significant predictor of elevated SBP in young adults, irrespective of gender. While BMI and WHR demonstrated strong associations with SBP in males, WC emerged as the most consistent predictor across both genders. These findings emphasize the importance of early monitoring of central adiposity, particularly WC, in young adults to mitigate the risk of hypertension and its cardiovascular complications.

By focusing on widely used anthropometric measures, this study offers practical insights for early hypertension risk detection and management. Early identification and intervention strategies tailored to central adiposity may help prevent hypertension progression and reduce long-term cardiovascular risks.
